# ALG: Automated Genotype Calling of Luminex Assays

**DOI:** 10.1371/journal.pone.0019368

**Published:** 2011-05-06

**Authors:** Mathieu Bourgey, Mathieu Lariviere, Chantal Richer, Daniel Sinnett

**Affiliations:** 1 Sainte-Justine Hospital Research Center, University of Montreal, Montreal, Quebec, Canada; 2 Department of Pediatrics, Faculty of Medicine, University of Montreal, Montreal, Quebec, Canada; Institute Biomedical Research August Pi Sunyer (IDIBAPS) - Hospital Clinic of Barcelona, Spain

## Abstract

Single nucleotide polymorphisms (SNPs) are the most commonly used polymorphic markers in genetics studies. Among the different platforms for SNP genotyping, Luminex is one of the less exploited mainly due to the lack of a robust (semi-automated and replicable) freely available genotype calling software. Here we describe a clustering algorithm that provides automated SNP calls for Luminex genotyping assays. We genotyped 3 SNPs in a cohort of 330 childhood leukemia patients, 200 parents of patient and 325 healthy individuals and used the Automated Luminex Genotyping (ALG) algorithm for SNP calling. ALG genotypes were called twice to test for reproducibility and were compared to sequencing data to test for accuracy. Globally, this analysis demonstrates the accuracy (99.6%) of the method, its reproducibility (99.8%) and the low level of no genotyping calls (3.4%). The high efficiency of the method proves that ALG is a suitable alternative to the current commercial software. ALG is semi-automated, and provides numerical measures of confidence for each SNP called, as well as an effective graphical plot. Moreover ALG can be used either through a graphical user interface, requiring no specific informatics knowledge, or through command line with access to the open source code. The ALG software has been implemented in R and is freely available for non-commercial use either at http://alg.sourceforge.net or by request to mathieu.bourgey@umontreal.ca

## Introduction

A single nucleotide polymorphism (SNP) is a DNA sequence variation that occurs at a single nucleotide position in the genome. As genotyping has become less expensive, it has become common to attempt to map disease genes via genome-wide scans [1]. Moreover, SNPs are the most commonly used polymorphic markers to identify candidate genes for complex diseases in genetic epidemiology studies [2], [3]. Genotyping errors are inherent to both family-based and case-control genetic association studies [4], [5], [6] and can lead to biased allelic and genotypic frequencies and thus either increases type I error rates [4], [7], [8] and decreases in power [9], [10]. In the case of candidate gene studies, the Luminex® 100/200 xMap technology (Austin, TX) is relatively inexpensive and easy to operate and maintain. With 100 separate identifiable beads available, a theoretical maximum of 50 different mutations can be assayed simultaneously on this platform [11]. This medium throughput SNP Genotyping system is ideal in clinical facilities for all sorts of genotyping applications, including pharmacogenomics [12], [13], [14] and medical genetic applications [15], [16] as well as population genetics [17], [18].

On the other hand, an important limitation of the Luminex genotyping platform is the lack of a freely available automated genotype calling software. The commercial STarStation/STarBase SNP or MasterPlex GT V2.3 analysis softwares can be purchased respectively from Applied Cytometry® (Sheffield, UK) and MiraiBio® (San Francisco, USA); otherwise, genotypes must be called manually, which could incur substantial increases in time and in genotype errors due to user subjectivity and human error. In response to the need for additional Luminex genotype calling software, we have developed the Automated Luminex Genotyping (ALG) software package that allows for extensive genotype calling from Luminex assays using either a friendly graphical user interface (GUI) or a command line interface in R. As we describe here, the ALG software is efficient and provides internal quality controls, and is an ideal alternative to the current commercial software. These properties have been confirmed by the blind analysis of a childhood leukemia dataset.

## Results and Discussion

**Figure 1:**
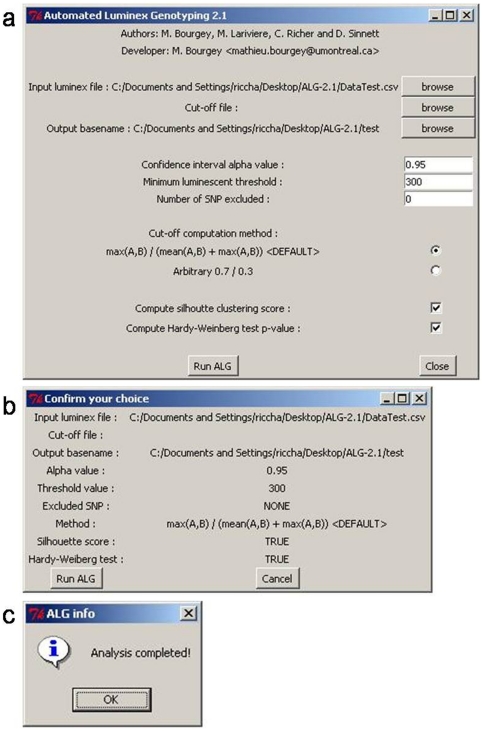
Graphical user interface provided in ALG. The Graphic User Interface (GUI) provided in the Automated Luminex Genotyping software (ALG) allows effective management of the genotype calling process. The main interface of the GUI (a) is dedicated to input and output file determination and to parameter selection. The confirmation interface (b) is used to verify parameter selection and to run the ALG analysis. The final interface (c) informs the user of analysis completion.

**Figure 2:**
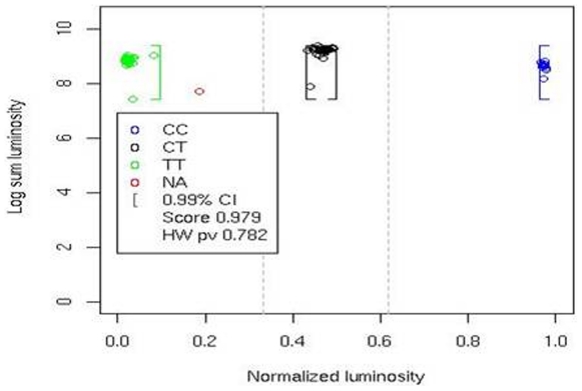
Graphical plot of data clustering. Data representation is given as a plot of sum of luminosity on a log scale as a function of the normalized luminosity for a C/T SNP genotyped in 91 individuals. The X axis represents the normalized intensity ϕ, whereas the Y axis represents the sum of the mean intensities of both probes, on a log scale. Each point represents an individual genotype and points are clustered in three groups based on genotype: CC (blue); CT (black); TT (green); no-calls are shown in red. The brackets represent the confidence interval boundaries for a type 1 error of 0.01. The silhouette score (0.979) and HWE p-value (0.782) are also reported.

ALG was used to genotyped a set of 95 SNPs in a cohort consisting of 300 childhood acute lymphoblastic leukemia patients and 329 healthy controls from the province of Quebec. Of these, 84 SNPs yielded distinct genotype clusters that were subsequently validated by manual inspection, providing a 88% SNP to assay conversion rate. We selected 3 SNPs based on the presence of independent sequence analysis (Sanger sequencing) in order to allow comparing genotypes obtained by ALG methods to those coming from the sequencing experiment considered as true genotypes. These 3 SNPs, rs2267437, rs828907 and rs11685387 were analyzed at blind. Genotypes were called twice, firstly in a process totally automated by ALG using defaults setting and secondly genotype calls were done manually. Manual calls can be easily made by adjusting settings of the software using the GUI (figure 1) based on graphical plot (figure 2) inspection of the data clustering. Two of the SNPs were also genotyped in two independent experiments to allow testing the reproducibility of calls. At the end the performance analysis consisted in approximately 9000 genotypes called.

**Figure 3:**
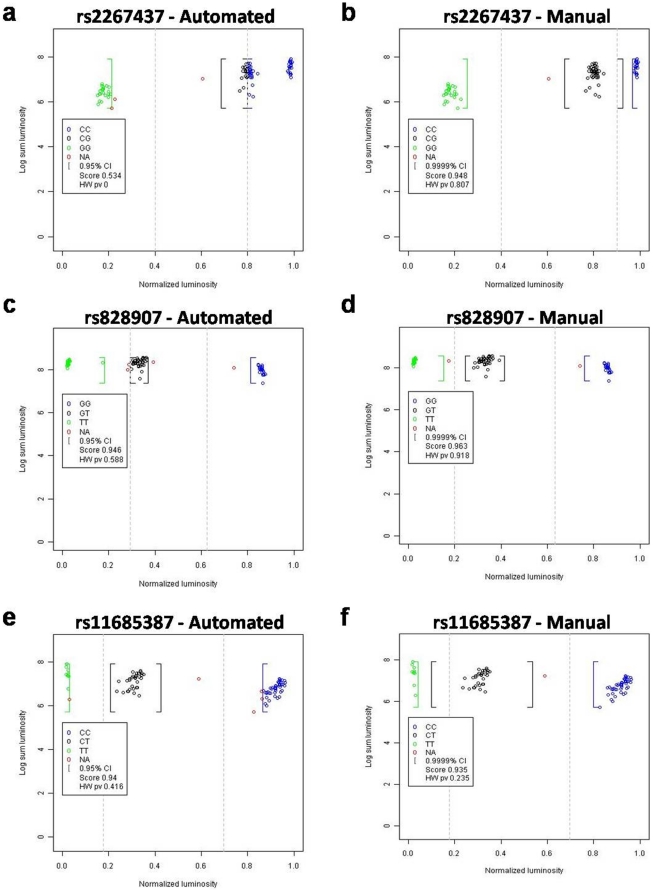
Manual versus automated genotype calls. An example of manual versus automated genotype calls obtained from the same assay is provided for SNP rs2267437 (a and b), rs828907 (c and d) and rs11685387 (e and f). The X axis represents the normalized intensity ϕ, whereas the Y axis value represents the logarithm of the sum, of the mean intensity of both probes. Automated calls (a, c and e) were obtained using the default parameters of ALG: confidence α = 0.05; minimum luminescent threshold  = 300 and default cut-off definition method. These parameters yield cut-off values of 0.8/0.4, 0.6/0.3 and 0.7/0.2 respectively for rs2267437, rs828907 and rs11685387. Manual calls (b, d and f) were obtained with the following user-defined parameters: confidence α = 0.0001; minimum luminescent threshold  = 300; default cut-off definition method. These parameters yield cut-off values of 0.6/0.3 and 0.7/0.2 respectively for rs828907 and rs11685387. Cut-off values for the SNP rs2267437 were set to 0.9/0.4 after visual inspection of the results.

Figure 3 provides an example of automated versus manual genotype calling experiment. The fully automated method (when the settings used are the default ones) is underestimating genotype: at 95% confidence interval the NA calls (corresponding to no call) are excessively high. Changing 95% to 99.99% had a huge impact on the number of no-calls. Manually overwriting the automatic cut-off for the SNP rs2267437 was necessary to get the proper genotyping. Decrease in the overall mean intensity below the minimal threshold could be influenced by the nucleotidic composition surrounding the SNP which in turn could reduce probe specificity. Chemistry of the beads in conjunction with specific sequence can give shifted MFI (mean fluorescence intensities) values. Nevertheless, a quick visual inspection of the graph provides enough flexibility to obtain robust genotypes out of these experiments. Table 1 gives a summary of the genotyping of ALL data. Using a manual management of parameters ALG reached an overall accuracy of 96.1% and a reproducibility of 94.1%. Here, the 4% of genotypes not correctly called is as a majority composed of genotypes that are not called (2.6% of NA and 0.8% of THR calls). Additionaly we compared 1100 calls made by ALG on these three SNPs to those obtained using the commercial MasterPlex GT suite® (MiraiBio Group of Hitachi Software Engineering America, Ltd. http://www.miraibio.com/masterplex-gt/gt-overview.html). MasterPlex software gave an overall accuracy of 89% whereas ALG reached 98% when the software is used with a manual management of parameters. Thus when a genotype is called by ALG, this call is highly accurate (99.6%) and reproducible (99.8%). These high levels of reproducibility prove the robustness of the method to experimental variations. Moreover they emphasized an important point where experimental variation that can not be caught by the method will lead to no calls and not to genotyping errors. Despite that manual setting gives more accurate results than the use of default settings, approximately 90% of results show similarity between the two settings. Globally default settings developed based on our experiments are sufficient to provide efficient calls (86.7% of reproducibility and 93.9% of accuracy). However, we recommend users to manually manage parameters: confidence level, threshold value and cut-off value. At the end of the call procedure, it is also possible to manually call unknown genotypes (no call). But once manual adjustments are defined for a SNP, they can be automatically re-used for the genotype call of other samples/plates for the same SNP. The relevance of genotypes called by ALG has been shown in a recent replication study [19].

**Table 1:** Summary of ALL data analysis.

Analysis information	Count	Overall	%
Default vs. Manual	4013	4473	89.7
Reproducibility 1 - Default	1004	1330	75.5
Reproducibility 1 - Manual	1251	1330	94.1
Reproducibility 2 - Default	986	1137	86.7
Reproducibility 2 - Manual	1234	1236	99.8
NA calls - Default	142	4473	3.2
NA calls - Manual	118	4473	2.6
THR calls - Default	220	4473	4.9
THR calls - Manual	37	4473	0.8
Accuracy 1 - Default	3860	4473	86.3
Accuracy 1 - Manual	4299	4473	96.1
Accuracy 2 - Default	3860	4111	93.9
Accuracy 2 - Manual	4299	4318	99.6

Results represent a pooled analysis of the 3 SNPs comparing calls made using either the default or manual settings of the software. The genotypes that can not be called are sub-divided in two classes: NA calls which correspond to an inappropriate normalized fluorescent value outside of confidence intervals and THR calls, which correspond to raw fluorescence under the minimal threshold. Accuracy, which represents the percent of ALG calls similar to the calls obtained by sequencing experiments, and reproducibility are measured in each condition. Reproducibility and accuracy are measured when all calls are taken into account (1) or when only genotypic calls are considered (2, no Na and THR calls). Measures are reported in terms of number of SNPs specifically called, the overall number of SNPs called and the corresponding percentage.

Actually no freely available software has been proposed to automate the genotype calling on the Luminex platform. In order to overcome this situation, we propose the accurate ALG clustering tool. ALG is semi-automated, requiring no prior manual inspection of the microassay Luminex data, and provides numerical measures of confidence for each SNP called, as well as an effective graphical plot (Figure 2) of the data clustering for visualization, optimization or troubleshooting purposes. As shown by genotypes calls in leukemia cohort, ALG is highly accurate, provides a very low no call threshold and performs very well when compare to commercial software. Note that no-calls are mainly affected by the confidence value and the cut –off definition. In some cases, poor DNA quality will increase the level of no-call. But in most cases, three well defined clusters were obtained leading to accurate genotype calls.

## Materials and Methods

### Study subject

We investigated ALG performance by genotyping the Quebec childhood acute lymphoblastic leukemia (ALL) cohort. The study population has been previously described [20], [21]. Our cohort includes 189 boys and 132 girls with a median age of 4.7 years, all French-Canadian from the province of Quebec, Canada. Parental DNA was available for 203 of the probands. Healthy controls (n = 329) consisted of French-Canadian individuals. The CHU Sainte-Justine Research Ethics Board approved the research protocol and written consent was obtained from all participants and/or their parents. DNA was isolated from buccal epithelial cells, peripheral blood or bone marrow in remission as previously described [22]. SNPs were genotyped using the Luminex xMAP/Autoplex Analyser CS1000 system (Perkin Elmer, Waltham, MA). Genetic variants were amplified using allele-specific primer extension in multiplexed assays and hybridized to Luminex MicroPlex TM –xTAG Microsperes as per Koo et al. [23]. Primer sequences for PCR amplification and for ASPE hybridization are given in Table S1 and S2 respectively. Amplification conditions and reaction conditions are available upon request.

### Implementation

Luminex assay analysis gives quantitative values that measure the mean intensities of fluorescence of each allele. So for a SNP marker with alleles A and B, which will give three possible genotypes: AA, AB and BB, the mean intensities are vA and vB. Individuals with genotype AA are expected to have high vA value and low vB value. By contrast, individuals with genotype BB are expected to have high vB value and low vA value. Individuals with genotype AB are expected to have similar vA and vB values. vA and vB values obtained for the 3 SNPs on a subset of the cohort are given in the Table S3. To facilitate the genotype call, we created a normalized value of intensity ϕ computed from vA and vB value. ϕ is the ratio of vA reported on the sum of vA and vB:
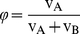
(A)


Individuals with genotype AA will have an ϕ close to 1; an ϕ close to 0 will correspond to individuals with BB genotype and individuals with AB genotype will have an ϕ of approximately 0.5. Following genotyping of a large amount of individuals, this approach will provide three clusters corresponding to pools of individuals with the same genotype.

In such case, genotyping will consist of defining the boundaries of each cluster and determining the individuals that belong to them. An accurate definition of clusters will allow unbiased genetic analyses. Ambiguous individuals located outside of clusters will be automatically considered as no call. Four main features could influence the clustering: (1) the cut-off definition allows groups to be created with similar ϕ values; (2) the definition of the group boundaries using the mean value and confidence interval method to determine call accuracy; (3) each group of calls must undergo quality controls to verify that there are no experimental or mathematical aberrations that have biased the calling procedure; (4) this approach must be applicable also to multi-allelic SNPs.

#### Defining the cut-offs

The cut-offs are two numerical values (CoD and CoU) that are used to separate the possible range of normalized values in three intervals: low values [0; CoD[, intermediates values [CoD; CoU[and high values [CoU; 1]. High values will allow assignment of AA genotypes, intermediate values for AB genotypes and low values for BB genotypes. The ALG software provides two methods to compute cut-off values. The first method is an arbitrary definition of the cut-offs where CoD is equal to 0.3 and CoU to 0.7. The principle behind this definition is to strictly follow the mathematical definition of genotypes and do not consider experimental variations. In this scenario one expects ϕAA values to be close to 1, ϕBB values to be close to 0 and ϕAB values to be around 0.5 in order for the intervals created by the arbitrary CoD and CoU values to correctly discriminate between the three groups.

However, if experimental variation occurs, then the position of three clusters can be skewed. For example a distribution of clusters, where ϕAA values are close to 1, ϕBB values are around 0.3 and ϕAB values are around 0.75, is possible if the probe of the A allele is more luminescent. In that case the theoretical definition of cut-offs (0.3 and 0.7) is not accurate. So we develop in ALG a second cut-off computation method that takes into account experimental variability. For each SNP, the cut-off computation is based on the maximum and the mean of the fluorescence intensities measured for the two alleles as such:
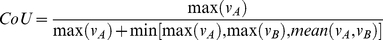


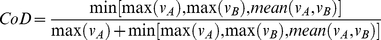



Max(vA) is the maximal vA value. The min[max(vA),max(vB), mean(vA,vB)] term represents the minimal value of either the maximal vA value, the maximal vB value or the mean value computed on all vA and vB values (mean(vA,vB)). For the usual situation in which all three genotypes are present, we expect that a sufficient proportion of vA values are higher than vB values (for AA genotypes) and a sufficient proportion of vB value are higher than vA values (for BB genotypes). In that case, the range of vA and vB is large and the mean of the overall value should be lower than the maximum of both vA and vB. So the min[max(vA),max(vB),mean(vA,vB)] value will correspond to mean(vA,vB) term. Thus cut-off values will be correlated to the ratio of the global mean reported on the maximum of vA (mean-max ratio). If measures are normally distributed and if probes has equivalent fluorescent level, this ratio will be close to 1/2 (the mean is half the maximum) and cut-off values close to 2/3 and 1/3 for CoU and CoD, respectively. In that case, the experimental based cut-off definition will approximate the arbitrary values (0.7 and 0.3). However, if experimental specificities provide variation in the mean-max ratio, the cut-off will follow this variation. For example, if the mean-max ratios are equal to 1/3, 2/3 or 1 the CoU values are 3/4, 3/5 and 1/2, respectively, showing the limitation of arbitrary values. Note that the most important factor that can influence the cut-off definition is the probe intensity balance, a variation in the strength of intensity between the two probes of a same SNP. In that case the default cut-off definition will provide the most accurate estimates. On the other hand this method will provide skewed cut-off values when genotyping monoallelic SNPs and in that case the theoretical definition of cut-off (0.7 vs 0.3) should be more appropriate. Finally, as experimental variation can be unpredictable, the software also includes the option of manually specifying the cut-off values by inputting a cut-off value file.

#### Defining the cluster boundaries

Using the two cut-off values (CoU and CoD), normalized mean of intensity values ϕ are separated into three groups: lower, median and higher. To ensure that clusters are accurately defined, we computed confidence intervals under a standard normal distribution using the following formula:
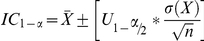



X represents the sample of intensities in each of the three groups formed by the two cut-off values, 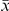 is the mean of X, σ is the standard deviation of X, n is the total sample size, α is the type I error rate and U_1-α/2_ is the standard normal value for the quantile 1-α/2. As the normalization step shown in equation A induces upper and lower limits of 0 to 1 for the range of ϕ values, confidence intervals used to define the clusters in each group have the same natural limits. Taking into account these limits, the lower boundary of the lower group (BB genotypes) is limited at 0 and the upper boundary of higher group (AA genotypes) is limited at 1. Therefore confidence intervals are measured only for one boundary of the AA group and for one boundary of the BB group, whereas both upper and lower confidence interval boundaries are computed for the median BA group. Thus we can define the cluster boundaries for each genotype using the confidence interval formula and removing the sample size term:
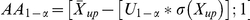


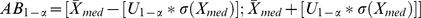


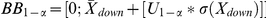



By default the type I error rate is 0.05, corresponding to the commonly used threshold of significance. However, users are allowed to modify α to reduce or increase the cluster stringency. Note that normalized intensities were approximated with a normal distribution. This implies that for analysis to be performed, certain conditions (such as independency and identical-distribution with finite variance) should be met. We considered that the conditions are met when the normalization was done on sample of a size higher or equal than 30 individuals for each SNP (the principle of the central limit theorem). For smaller sample size, normalized intensities will not follow a normal distribution implying that both cut-offs and confidence intervals could not be applicable. In that case, ALG software will still provide genotyping calls but they will come with warning messages that inform users of the possible non-normality of the data.

#### Quality controls

We further developed the software to control for quality of genotype calls. ALG currently offers six quality control tools. First, ALG controls the minimum threshold of fluorescence, which represents the minimum measure for each individual to be considered as a potential call. For a given SNP, if the sum of the mean intensity for each probe is lower than a minimum threshold of fluorescence, then the genotype calling of these individuals will not be considered in the analysis and the value THR (for minimum threshold) is returned as call. Based on experimental tests the minimal threshold has been fixed to 300 by default; however users are allowed to adjust it in function of their specific experimental conditions.

The next quality control tool of ALG is used only when more than 80% of individuals are found in the same cluster. Using this tool, ALG has the power to investigate whether experimental artefact has biased the analyses. To do so, we perform a second round of computations of the cut-off values and confidence interval boundaries using only the individuals found in this cluster. Clustering of these individuals validates the first round of computation, which indicates that the initial clustering was correct. Grouping of the individuals in different clusters shows that individuals are not homogeneous and need to be divided into different groups. This cancels the first analysis and we continue the analysis using the second cut-off values. New confidence interval boundaries are computed using these new cut-off values and all the individuals used for the first analysis.

ALG also contains mathematical quality control tools. During the analysis, it is important to avoid overlap in different clusters. Overlapping cluster will result in two different genotypes for an individual and thus lead to a no call genotype. In case of overlap, the boundaries of the two clusters are moved to create flanking confidence intervals. The break point between the two clusters is determined as the point located at equidistance from the old overlapping boundaries. Limiting the cut-off deviation from the expected 0.3 and 0.7 values is crucial for the analysis. To consider the presence of three possible clusters, the CoU is limited to values higher than 0.6 and CoD to values lower than 0.4. These mathematical controls are essential in order to both limit the accumulation of no calls and to avoid extreme values created by experimental variation that can bias the analysis.

Finally, the software has quality control tools that are post calling controls that verify the robustness of the genotype calls. These controls correspond to the computation of both the Silhouette scores [24] and Hardy-Weinberg equilibrium tests. Using a Silhouette calculation, we can determine the distance from each data point in a cluster to all other data points within the same cluster and to all data points in the closest cluster. Thus this calculation provides a measure of how well a data point is classified when it is assigned to a given cluster according to both the tightness of the clusters and the separation between them [25]. The Silhouette score condenses the cluster quality for each SNP assay into a single measure that ranges from 1 to -1. It is recommended to accept the results from SNP assays with Silhouette scores >0.65, to fail the whole assays if the Silhouette scores is <0.25 and to manually inspect the assay results if the score is included in the [0.25; 0.65] interval. ALG also performs a Hardy-Weinberg equilibrium test. This test is a Pearson's chi-squared test, using the observed genotype frequencies obtained from the genotype calling and the expected genotype frequencies under Hardy-Weinberg proportions. The quality of the data is reported in terms of type I error. As the Hardy-Weinberg equilibrium test could be biased in the case where SNPs chosen to be genotyped are correlated to the sample ascertainment, the Hardy-Weinberg quality controls is only given for information purpose and the corresponding rejection of SNPs must results from detailed inspection of the data and of the assay design. Note that a bias in Hardy-Weinberg equilibrium is possible when one tries to genotype SNPs associated to a disease in a set of individuals that have develop the disease. In which case, we would expect the genotype distribution in individuals not to follow the Hardy-Weinberg proportion due to the correlation between the SNPs and the disease. Thus, to reduce this possible bias we recommend mixing cases and controls in the same assay.

#### Multiple alleles

**Figure 4:**
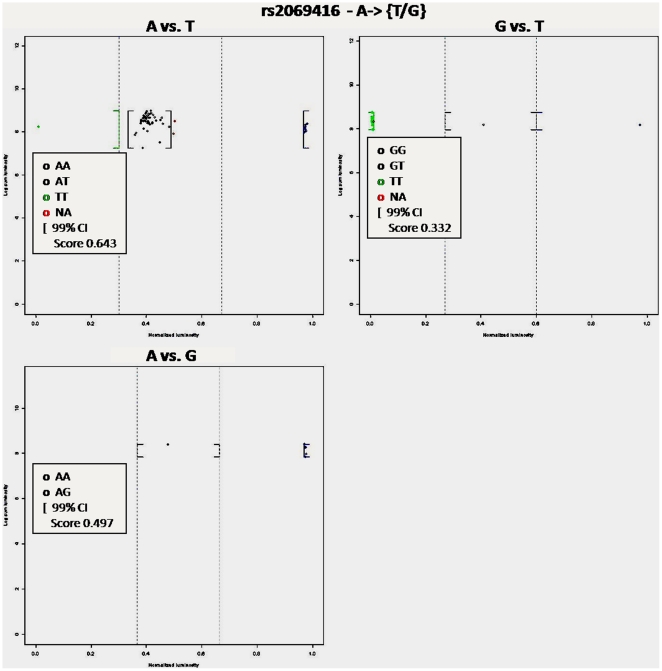
Genotype calls of the multi-allelic SNP rs2069416. ALG analysis of the multi-allelic SNP rs2069416. SNP rs2069416 has three alleles A, T and G leading to three independent genotype calling procedures: A vs T, A vs G and G vs T. The procedure in which an individual is analyzed depends on its two most luminescent probes. The X axis represents the normalized intensity ϕ, whereas the Y axis value represents the sum of the mean intensity of both probes on the log scale. Genotype calls are obtained using user-specific parameters of ALG: confidence α = 0.05; minimum luminescent threshold  = 200; default cut-off definition method. These parameters give cut-off values of 0.6/0.3 and 0.7/0.4 respectively for A vs G and G vs T procedures. Cut-off values for the A vs T were set to 0.7/0.3 after visual inspection of the results.

An important limitation in most genotype calling algorithms is dealing with multi-allelic SNPs. Ignoring multi-allelic SNPs could leads to bias genetic association studies [26]. Multi-allelic SNPs represent approximately 1% of SNPs found in Ensembl release 43. In ALG multi-allelic SNPs are processed in a very simple manner. ALG creates subgroups of individuals based on the two most fluorescent probes. Then each subgroup is analyzed based on genotyping a biallelic SNP. For example, if one genotype a SNP with three alleles A, B and C, ALG creates three subgroups of individuals: 1) individuals who are analyzed for alleles A and B (genotypes AA, AB and BB); 2) individuals who are analyzed for alleles A and C (genotypes AA, AC and CC) and 3) individuals who are analyzed for alleles B and C (genotypes BB, BC and CC). Figure 4 shows an example of genotype calls for the multi-allelic SNP rs2069416. This simple method to deal with multi-allelic SNPs is very accurate because each individual carried only two alleles and probes other than the two most fluorescent ones can be consider as background noise. As multiple allele procedure does not consider the entire set of individuals in the same analysis, Hardy-Weinberg quality control was not applied. Note that the method is limited by the minimal sample size (30 individuals) required for each sub-group to stay under the normality assumption that validate cut-offs and confidence intervals computation.

### Availability and Future Directions

Additional improvement should be done to both increase the efficiency of software and reduce the manual management of the software parameters. The high efficiency of the ALG algorithm, proven by the analysis of real data, makes also conceivable to adapt it to other genotyping platforms. The ALG package provides an implementation of this tool in the open source R programming environment that will promote additional development either by actual developers or by external users. Finally the ALG GUI allows a user friendly interface to input data and run analyses without specific informatics knowledge. ALG is actually available for both windows and Linux/Unix operating systems at the project home page: http://sourceforge.net/projects/alg.

## Supporting Information

Table S1 **PCR amplification primers.** The PCR amplification primers and the size of the fragment generated using these primers are given for the 3 SNPs included in the performance analysis.
(DOCX)

Table S2 **ASPE hybridization primers.** The ASPE hybridization primers and the corresponding bead number on the assay are given for the 3 SNPs included in the performance analysis. The lowercase sequence represents the part of the primer sequence specific to the bead whereas the uppercase sequence characterises the SNP locus. The SNP allele corresponds to the last base of the primer sequence represented here in bold. * indicates uppercase sequences designed from the reverse strand.
(DOCX)

Table S3 **Median FMI values and genotype call.** The median MFI values obtain from the Lunminex analysis with standard sensitivity are provided for the 3 SNPs included in the performance analysis. The values are available for a subset of 38 control individuals. For each SNP and each individual the median MFI values are given for both the reference and the variant probes. The corresponding individual's genotype determined by the ALG software is represented through the background colour of the cells. Blue cells indicate homozygote reference calls, pink cells represents homozygote variant calls and orange cells correspond to heterozygote calls. White cells are used to identify no-calls (THR; NA call or blank negative controls).
(DOCX)
