# Supplementary material for: ALG: Automated Genotype Calling of Luminex Assays
Source: PLoS One. 2011 May 6;6(5):e19368. doi: 10.1371/journal.pone.0019368 (PMC3089621; doi:10.1371/journal.pone.0019368)
Supplement: Table S1 — PCR amplification primers. The PCR amplification primers and the size of the fragment generated using these primers are given for the 3 SNPs included in the performance analysis. (DOCX) [file pone.0019368.s001.docx]

| SNP | Primer | Size (bp) |
| --- | --- | --- |
| rs2267437 | TCGCCACGGGTCCCAATGGA | 447 |
|  | GCGCCAAGGAGGCAGGAGTG |  |
| rs828907 | TGCCGAGCTGATCTTTCTCT | 207 |
|  | GAGGTGCTCATAGAGCCCAG |  |
| rs11685387 | GAGCTCTGACACAAAATGCCT | 164 |
|  | ATGCGCACATTCTCTCCATT |  |
