# Supplementary material for: ALG: Automated Genotype Calling of Luminex Assays
Source: PLoS One. 2011 May 6;6(5):e19368. doi: 10.1371/journal.pone.0019368 (PMC3089621; doi:10.1371/journal.pone.0019368)
Supplement: Table S2 — ASPE hybridization primers. The ASPE hybridization primers and the corresponding bead number on the assay are given for the 3 SNPs included in the performance analysis. The lowercase sequence represents the part of the primer sequence specific to the bead whereas the uppercase sequence characterises the SNP locus. The SNP allele corresponds to the last base of the primer sequence represented here in bold. * indicates uppercase sequences designed from the reverse strand. (DOCX) [file pone.0019368.s002.docx]

| SNP | Primer | Bead number |
| --- | --- | --- |
| rs2267437 | tacatcaacaattcattcaatacaGGCCAGAGGGTGGCG**C*** | 46 |
|  | cttttacaatacttcaatacaatcGGCCAGAGGGTGGCG**G*** | 20 |
| rs828907 | caatttcatcattcattcatttcaTACCCGCAGGGAAGGGC**G** | 35 |
|  | caattcatttcattcacaatcaatTACCCGCAGGGAAGGGC**T** | 36 |
| rs11685387 | tcaattacttcactttaatcctttCTTTAAGTAAATCTGTCGTACTAG**C** | 33 |
|  | tcattcatatacataccaattcatCTTTAAGTAAATCTGTCGTACTAG**T** | 34 |
