# Supplementary material for: ALG: Automated Genotype Calling of Luminex Assays
Source: PLoS One. 2011 May 6;6(5):e19368. doi: 10.1371/journal.pone.0019368 (PMC3089621; doi:10.1371/journal.pone.0019368)
Supplement: Supplementary file 3 [file pone.0019368.s003.docx]

SNP	rs2267437		rs828907		rs11685387	
**Allele**	**Reference**	**Variant**	**Reference**	**Variant**	**Reference**	**Variant**
**A110**	350	1396.5	146	5165	1490	93
**A118**	782	215	2670	4508	815	1836
**A121**	42.5	1920.5	4128	864.5	815	1754
**A124**	557	136	2852.5	4731.5	916	1739
**A129**	668	197	2133	3695	768	1384.5
**A130**	800	199	2548	4491	1655	66.5
**A132**	664.5	175	175.5	5717	1487	93.5
**A133**	220	683	2129	353.5	911	64
**A134**	12.5	26.5	289	927.5	288	804
**A135**	707	164	166	5405.5	1467.5	95
**A141**	37	2182	3583	726.5	1394	87.5
**A145**	756.5	203	149	5146	1628	78
**A146**	350.5	1257	3217	631	1375	81.5
**A147**	730	215	2289	3862.5	1694	91.5
**A151**	157	577	79	3319	981	57
**A152**	386	1638	3421	675	757.5	1432.5
**A154**	789	221	144	5286	1674	86
**A155**	417	1642	2441	4085	1482.5	90.5
**A157**	806	217	154.5	5561	1666	63
**A159**	657	180.5	3466	628	747	1376.5
**A161**	302	1174	3485	689	1483	86
**A165**	675	184	3218.5	617	761	1422
**A168**	875.5	214	3724	824	989	1726
**A169**	427	1498	2464	4393	841.5	1647
**A172**	834	203	2443	4139	809	1599
**A176**	404	1562	3238	591	53	2471.5
**A179**	825	194	1963.5	3409	1691	71
**A180**	671	208	2167	3638.5	779.5	1361.5
**A181**	402.5	1563	2513	4144	824	1482.5
**A183**	477	1622	3040	4424	1723	131.5
**A186**	37	2791	2169	3626.5	1405	62.5
**A189**	478.5	1617	3726.5	690	861	1624
**A190**	454	1636	2558	4277	840	1650
**A193**	1010	248	2881	4454	986	1843
**A194**	467	1696	137	5179	1795	114
**A196**	707	196	3503	649	1553	47
**A199**	53	3060	2539	4283	1746	49
**A200**	529	2094.5	4224	885	1095	2061
